# Social isolation and all-cause mortality: a population-based cohort study in Denmark

**DOI:** 10.1038/s41598-018-22963-w

**Published:** 2018-03-16

**Authors:** Kristina Laugesen, Lisbeth Munksgård Baggesen, Sigrún Alba Jóhannesdóttir Schmidt, M. Maria Glymour, Mathias Lasgaard, Arnold Milstein, Henrik Toft Sørensen, Nancy E. Adler, Vera Ehrenstein

**Affiliations:** 1 Department of Clinical Epidemiology, Aarhus University Hospital, Aarhus, 8200 Denmark; 2 Department of Epidemiology and Biostatistics, University of California, San Francisco, CA USA; 3 Central Denmark Region, DEFACTUM, Aarhus, 8200 Denmark; 4 Department of Psychology, University of Southern Denmark, Odense, 5230 Denmark; 5 The Clinical Excellence Research Center (CERC), Stanford University School of Medicine, Stanford, CA USA; 6 Department of Health Research & Policy, Stanford University, Stanford, CA USA; 7 Departments of Psychiatry and Pediatrics, University of California, San Francisco, CA USA

## Abstract

Social isolation is associated with increased mortality. Meta-analytic results, however, indicate heterogeneity in effect sizes. We aimed to provide new evidence to the association between social isolation and mortality by conducting a population-based cohort study. We reconstructed the Berkman and Syme’s social network index (SNI), which combines four components of social networks (partnership, interaction with family/friends, religious activities, and membership in organizations/clubs) into an index, ranging from 0/1 (most socially isolated) to 4 (least socially isolated). We estimated cumulative mortality and adjusted mortality rate ratios (MRR) associated with SNI. We adjusted for potential important confounders, including psychiatric and somatic status, lifestyle, and socioeconomic status. Cumulative 7-year mortality in men was 11% for SNI 0/1 and 5.4% for SNI 4 and in women 9.6% for SNI 0/1 and 3.9% for SNI 4. Adjusted MRRs comparing SNI 0/1 with SNI 4 were 1.7 (95% CI: 1.1–2.6) among men and 1.6 (95% CI: 0.83–2.9) among women. Having no partner was associated with an adjusted MRR of 1.5 (95% CI: 1.2–2.1) for men and 1.7 (95% CI: 1.2–2.4) for women. In conclusion, social isolation was associated with 60–70% increased mortality. Having no partner was associated with highest MRR.

## Introduction

Humans are highly social beings and social relations have influence on health. Former meta-analyses estimate a 50% improved survival associated with strong social relations^1^ and about 30% increase in mortality associated with both subjective and objective social isolation^2^. This evidence indicates that social isolation is an important risk factor for death in line with other well-known risk factors, such as obesity, inactive lifestyle and alcohol consumption^3^.

In 1979, Berkman and Syme constructed the Berkman and Syme’s social network index (SNI) as an objective measure of social relations and conducted a study on its association with all-cause mortality^4^. The SNI combines in a single score information on partnership, interaction with family and friends, religious activities, and membership in organizations and clubs. They found that poor social relations were associated with a two- to three-fold increased mortality regardless of health status, various lifestyle habits, obesity, socioeconomic status and health care use^4^. In contrast, others have investigated various pathways linking social isolation to increased mortality, suggesting that the association is indeed mediated at least partly through behavioral, psychological and biological factors^5–7^.

In a recent study from the U.S, Pantell *et al*. examined the association between social isolation and mortality with data from a National Health and Nutrition Examination Survey (NHANES), using the SNI^3^. In addition, they investigated which components of the SNI that were most predictive for mortality. Infrequent religious activity was associated with the greatest (nearly 30%) increase in mortality, surpassing the effect of absence of a partner (nearly 20%).

We aimed to investigate the effects of social isolation on all-cause mortality in a cohort design, using data from a population-based health survey in Denmark. We focused on which components of social relations (SNI components) that were the most important predictors for mortality, and how associations might vary across gender. Several studies have investigated the association between social isolation and mortality^1,2^. Meta-analytic results, however, indicate extensive heterogeneity in effect sizes across studies and found that studies with more covariate control had significantly lower effect estimates than studies with limited confounder adjustment. These results indicate the importance of evaluating associations between social connections and mortality in diverse settings with more comprehensive measures of potential confounders. In this study, we were able to incorporate adjustment for several major predictors of mortality, including underlying somatic and psychiatric morbidity, socioeconomic status and lifestyle factors, as potential confounders of the association. Among important factors are also the structure of a country’s social support and healthcare system. Although, the association between social isolation and mortality has been studied in multiple and diverse settings, including Scandinavian countries^8–10^, Denmark has an abundant social support system and free access to health care and education, and socioeconomic status is relative equally distributed in the population compared to other high-income countries^11–13^.

## Results

We included 21,604 participants of the “*Hvordan har du det?*” (HHDD, Danish for “How are you?”) survey^14^, of whom 53% were women. Mean (standard deviation) age was 52 (14) years for men and 51 (14) years for women. Persons with the lowest (most socially isolated) and with the highest SNI (least socially isolated) accounted for, respectively 10% and 11% of the respondents (Table 1). Men accounted for 57% of respondents with SNI of 0/1 and for 42% of the respondents with SNI of 4. High SNI was associated with higher probability of high-school level education, lower prevalence of current smoking, lower alcohol consumption, better self-rated health and lower medically-confirmed morbidity (Table 1). Regarding the individual SNI components; 3,398 (14%) were not in a marriage or steady partnership; 3,853 (22%) did not have frequent social contacts; 16,888 (81%) did not attend frequent religious participation; and 4,324 (23%) did not have frequent memberships in groups. Detailed distribution of the SNI variables is provided in the Supplementary Table S1.

**Table 1:** Characteristics of the study population.

	Social Network Index					Total
	0/1	2	3	4	Missing	
	N (%)	N (%)	N (%)	N (%)	N (%)	N (%)
**Characteristics**						
**Total**	2,143	6,768	10,224	2,429	40	21,604
**Gender**						
Women	927 (43)	3,430 (51)	5,705 (56)	1,404 (58)	19 (49)	11,485 (53)
Men	1,237 (57)	3,338 (49)	4,519 (44)	1,025 (42)	20 (51)	10,119 (47)
**Age category, years**						
25–39	350 (16)	1,605 (24)	2,777 (27)	421 (17)	3 (7.7)	5,156 (24)
40–59	1,015 (47)	3,127 (46)	4,685 (46)	978 (40)	10 (26)	9,815 (45)
60–79	779 (36)	2,036 (30)	2,762 (27)	1,030 (42)	26 (67)	6,633 (31)
**Minimum education, years**						
Missing	75 (3.5)	84 (1.2)	43 (0.4)	7 (0.3)	37 (95)	246 (1.1)
≤7	583 (27)	1,311 (19)	1,505 (15)	520 (21)	2 (5.1)	3,921 (18)
8–9	379 (18)	1,107 (16)	1,429 (14)	281 (12)	0 (0)	3,196 (15)
10	605 (28)	1,951 (29)	2,969 (29)	645 (27)	0 (0)	6,170 (29)
High school/Equivalent (minimum 12–13 years of education)	398 (19)	1,939 (29)	3,617 (35)	781 (32)	0 (0)	6,735 (31)
Other (e.g. skilled manual work)	104 (5.0)	376 (6.0)	661 (6.5)	195 (8.0)	0 (0)	1,336 (0.06)
**Personal annual income, Danish Kroner**						
Missing	218 (10)	508 (7.5)	699 (6.8)	179 (7.4)	31 (80)	1,635 (7.6)
0–99,000	182 (8.5)	574 (8.5)	809 (7.9)	308 (13)	0 (0)	1,873 (8.7)
100,000–149,000	453 (21)	1,026 (15)	1,161 (11)	352 (15)	3 (7.7)	2,995 (14)
150,000–249,000	562 (26)	1,851 (27)	2,810 (28)	656 (27)	2 (5·1)	5,881 (27)
250,000–374,000	520 (24)	1,887 (30)	3,096 (30)	615 (25)	2 (5·1)	6,120 (28)
375,000–524,000	142 (6.6)	648 (9.6)	1,146 (11)	233 (9.6)	0 (0)	2,169 (10)
≥525,000	67 (3.1)	274 (4.0)	503 (4.9)	86 (3.5)	1 (2.6)	931 (4.3)
**Smoking status**						
Missing	113 (5.3)	244 (3.6)	267 (2.6)	99 (4.1)	27 (69)	750 (3.5)
Never smoker	674 (31)	2,530 (37)	4,605 (45)	1,223 (50)	6 (15)	9,038 (42)
Former smoker	466 (22)	1,711 (25)	2,656 (26)	648 (27)	3 (7.7)	5,484 (25)
Current smoker	891 (42)	2,283 (34)	2,696 (26)	459 (19)	3 (7.7)	6,332 (29)
**Level of alcohol use**						
Missing	348 (16)	652 (10)	656 (6.4)	211 (8.7)	29 (74)	1,896 (8.8)
Low	1,235 (58)	4,360 (64)	7,110 (70)	1,716 (71)	8 (21)	14,429 (67)
Medium	358 (17)	1,325 (20)	1,918 (19)	404 (17)	0 (0)	4,005 (19)
High	203 (10)	431 (6·4)	540 (5.3)	98 (4.0)	2 (5.1)	1,274 (5.9)
**Body mass index**						
Missing	84 (3.9)	153 (2.3)	166 (1.6)	38 (1.6)	34 (87)	475 (2.2)
Underweight	53 (2.5)	116 (1.7)	116 (1.1)	27 (1.1)	0 (0)	312 (1.4)
Normal	958 (45)	3,212 (48)	5,044 (49)	1,136 (47)	1 (2.6)	10,351 (48)
Overweight	696 (33)	2,329 (34)	3,588 (35)	899 (37)	3 (7.7)	7,515 (35)
Obese	353 (17)	958 (14)	1,310 (13)	329 (14)	1 (2.6)	2,951 (14)
**Self-rated health**						
Missing	15 (0.7)	46 (0.7)	63 (0.6)	17 (0.7)	2 (5.1)	143 (0.7)
Excellent	150 (7.0)	593 (8.8)	1,185 (12)	227 (9.3)	3 (7.7)	2,158 (10)
Very good	525 (25)	2,367 (35)	4,327 (42)	1,005 (41)	9 (23)	8,233 (38)
Good	924 (43)	2,722 (40)	3,597 (35)	950 (39)	12 (31)	8,205 (38)
Not quite good	418 (20)	885 (13)	909 (8.9)	206 (8.5)	9 (23)	2,427 (11)
Bad	112 (5.2)	155 (2.3)	143 (1.4)	24 (1.0)	4 (10)	438 (2.0)
**Hypertension**	521 (24)	1,469 (22)	2,011 (20)	592 (24)	7 (18)	4,600 (21)
**COPD, bronchitis, emphysema**	67 (3.1)	113 (1.7)	115 (1.1)	28 (1.2)	0 (0)	323 (1.5)
**Diabetes**	107 (5.0)	237 (3.5)	253 (2.5)	82 (3.4)	2 (5.0)	681 (3.2)
**Dyslipidemia**	255 (12)	674 (10)	863 (8.4)	286 (12)	5 (13)	2,083 (9.6)
**Charlson comorbidity index**						
0	1,786 (83)	5,979 (88)	9,316 (91)	2,147 (88)	30 (77)	19,258 (89)
1–2	300 (14)	666 (9.8)	800 (7.8)	251 (10)	6 (15)	2,023 (9.4)
>2	58 (2.7)	123 (1.8)	108 (1.1)	31 (1.3)	3 (7.7)	323 (1.5)
**Antidepressant use**						
None	1,691 (79)	5,785 (86)	9,016 (88)	2,113 (87)	31 (80)	18,636 (86)
Past	251 (12)	579 (8.6)	726 (7.1)	175 (7.2)	2 (5.0)	1,733 (8.0)
Current	202 (9.4)	404 (6.0)	482 (4.7)	141 (5.8)	6 (15)	1,235 (5.7)
**Strong analgesics use**						
None	1,675 (78)	5,547 (82)	8,553 (84)	2,008 (83)	28 (72)	17,811 (82)
Past	361 (17)	968 (14)	1,374 (13)	348 (14)	10 (26)	3,061 (14)
Current	108 (5.0)	253 (3.7)	297 (2.9)	73 (3.0)	1 (2.6)	732 (3.4)

Level of alcohol use (Danish units of alcohol per week. One Danish unit of alcohol correspond to 15 millilitres or 12 gram pure alcohol): Low: <7 for women/<14 for men. Medium: 7–14 for women/14–21 for men. High: >14 for women/>21 for men. Body mass index: Underweight: <18.5 kg/m^2^. Normal weight: 18.5 −<25.0 kg/m^2^. Over weight: 25.0 −<30 kg/m^2^. Obese: ≥30 kg/m^2^. Antidepressant use: Current: ≤90 days before baseline. Past: >90 days before baseline. Strong analgesics use: Current: ≤90 days before baseline. Past: >90 days before baseline.

**Figure 1:**
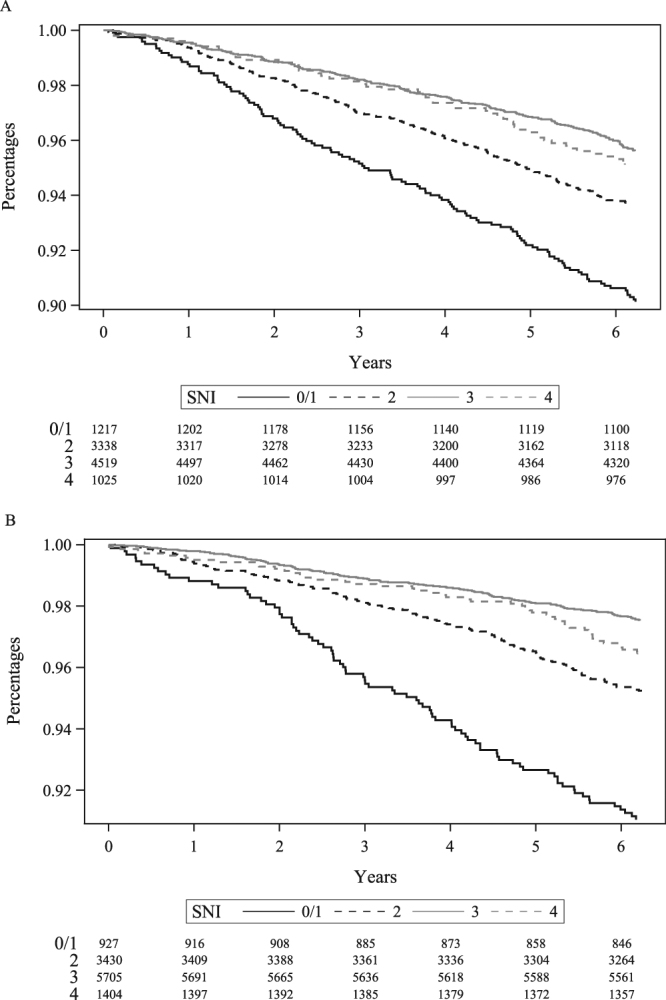
Crude Kaplan-Meier survival curves for all-cause mortality, stratified by Berkman and Syme’s social network index (SNI) and gender. (**A**) Men. (**B**) Women.

Crude cumulative mortality after seven years of follow-up was 11% in men with SNI 0/1, 6.9% in men with SNI 2, 4.9% in men with SNI 3, and 5.4% in men with SNI 4. For women, cumulative mortality was 9.6% for SNI 0/1, 5.1% for SNI 2, 2.8% for SNI 3 and 3.9% for SNI 4. Kaplan Meier survival curves are presented in Fig. 1, stratified by gender. Crude MRRs comparing SNI 0/1 with SNI of 4 were 2.4 (95% CI: 1.7–3.5) among men and 2.7 (95% CI: 1.8–4.0) among women. Adjusting for age, education and personal income (Model 1) did not affect the estimates (Table 2). Additional adjustment for lifestyle habits attenuated effect estimates [MRR in men 1.9 (95% CI: 1.2–2.9) and in women 1.9 (95% CI: 1.1–3.5)]. Further adjustment for medically-confirmed morbidity did not affect the previously adjusted estimates materially (Table 2). Crude MRRs comparing SNI 2 with SNI 4 was 1.4 (95% CI: 0.98–1.9) for men and 1.4 (95% CI: 0.96–2.0) for women, however the estimates were close to the null after adjustment for all covariates (Table 2). When changing the order of covariates in model 2 and 3 (i.e. introducing morbidity in model 2 and lifestyle in model 3), adjustment for morbidity attenuated the effect estimates [MRR in men 1.9 (95% CI: 1.3–2.9) and in women 2.2 (95% CI: 1.4–3.5) comparing SNI 0/1 with SNI 4] (Supplementary Table S2, model 2). Nevertheless, lifestyle still accounted for some of the confounding (Supplementary Table S2, model 3). For the individual SNI components, MRR associated with having no partner were 1.4 (95% CI: 1.1–1.7) for men and 2.9 (95% CI: 2.3–3.7) for women, adjusting only for the remaining SNI components. Corresponding MRRs for lack of membership in clubs or organizations were 2.4 (95% CI: 1.9–2.9) among men and 1.9 (95% CI: 1.5–2.5) for women (Table 2). For women, the marriage/partner component effect was attenuated but not explained entirely by adjustment for the covariates [adjusted MRR in women 1.7 (95% CI: 1.2–2.4)], whereas the estimate for the clubs and organization component was close to the null after covariate adjustment. The remaining SNI components–contacts with friends and relatives and participation in religious activities–were not strongly associated with an increased mortality (Table 2).

**Table 2:** Association between social isolation and all-cause mortality in Denmark.

	Men				Women			
	MRR (95% CI)				MRR (95% CI)			
	Crude	Model 1	Model 2	Model 3	Crude	Model 1	Model 2	Model 3
Social network index (SNI)								
0/1 vs 4	2.4 (1.7–3.5)	2.4 (1.6–3.5)	1.9 (1.2–2.9)	1.7 (1.1–2.6)	2.7 (1.8–4.0)	3.0 (1.9–4.8)	1.9 (1.1–3.5)	1.6 (0.83–2.9)
2 vs 4	1.4 (0.98–1.9)	1.5 (1.1–2.2)	1.1 (0.76–1.7)	1.1 (0.74–1.7)	1.4 (0.96–2.0)	2.0 (1.3–3.1)	1.4 (0.81–2.3)	1.2 (0.73–2.1)
3 vs 4	0.95 (0.68–1.4)	1.3 (0.90–1.9)	1.2 (0.83–1.8)	1.3 (0.8–1·9)	0.77 (0.54-1.1)	1.3 (0.86–2.0)	1.0 (0.62–1.7)	0.98 (0.59–1.6)
SNI component (no vs. yes)								
Married/partner	1.4 (1.1–1.7)	1.5 (1.1–1.9)	1.6 (1.2–2.1)	1.5 (1.2–2.1)	2.9 (2.3–3.7)	2.1 (1.6–2.7)	2.1 (1.5–2.8)	1.7 (1.2–2.4)
Social contact	1.0 (0.82–1.3)	1.0 (0.81–1.3)	1.0 (0.76–1.3)	1.1 (0.82–1.4)	1.1 (0.83– 1.6)	1.2 (0.88–1.7)	1.3 (0.88–2.0)	1.2 (0.73–2.0)
Religious activities	0.75 (0.58–0.98)	1.1 (0.80–1.4)	1.0 (0.74–1.4)	1.0 (0.73–1.4)	0.57 (0.44–0.74)	1.0 (0.79–1.4)	0.77 (0.54–1.1)	0.77 (0.52–1.1)
Clubs/organizations	2.4 (1.9–2.9)	1.6 (1.3–2.0)	1.3 (1.0–1.7)	1.2 (0.89–1.5)	1.9 (1.5–2.5)	1.6 (1.2–2.1)	1.2 (0.87–1.8)	1.1 (0.70–1.6)

Model 1: adjusted for age, education, and income.

Model 2: adjusted for age, education, income, smoking, alcohol, body mass index, and regular exercise.

Model 3: adjusted for age, education, income, smoking, alcohol, body mass index, regular exercise, self-rated health, Charlson Comorbidity Index, past or present use of antidepressants, and past or present use of strong analgesics.

In the regression models, we accounted for survey design and non-response by using post-survey weight.

In sensitivity analyses, we found similar estimates when stratifying on income level indicating no effect measure modification of income (Supplementary Table S3). Second, when evaluating reverse causation, the estimates did not change substantially after excluding persons with high level of comorbidity (Charlson Comorbidity Index >2) or persons with terminally illness (Supplementary Table S4).

## Discussion

In a large population cohort of Danish adults followed for up to 7 years, social isolation was associated with an increased all-cause mortality. After adjustment for socioeconomic status, lifestyle factors and psychiatric and somatic morbidity, mortality was increased by 1.7 and 1.6-fold in men and women when comparing most socially isolated to least socially isolated people. Among the different components of social relations (marriage/partnership, social contacts, religious participation and membership in clubs/organizations), not having a spouse or a partner compared to being in a marriage/partnership was associated with highest MRR.

Our study supports previous evidence of social isolation being a predictor of mortality^2–4^. However, former studies report heterogeneity in effect sizes, which may be explained by different populations and settings, different methodology or different confounder control. The U.S study by Pantell *et al*.^3^ used the same measure of social network and had very similar confounder control as our current study, wherefore the two studies are comparable. They found similar increase in mortality when comparing the most socially isolated (SNI 0/1) with the least socially isolated (SNI 4). In our study, cumulative mortality was highest in SNI 0/1 followed by SNI 2 and lowest in SNI 3 and 4. The cumulative mortality were very similar for SNI 3 and 4. A similar effect was observed in the crude relative estimates, although attenuated after adjustment. Our results are in line with former aggregated data suggesting more of a continuum than a threshold at which risk becomes pronounced^2^. In our study, not having a spouse or a partner was associated with highest MRR and 14% of our study population were not married or in a steady partnership. The association between marital status and mortality has been confirmed in prior studies^3,10,15–18^. In Pantell *et al*., infrequent religious activity was associated with the greatest (nearly 30%) increase in mortality, surpassing the effect of absence of a partner^3^. Associations between religious activity and lower all-cause mortality have been shown in Danish populations^19,20^. Our study confirms this finding, although the association attenuated after adjusting for covariates. In our study, 81% did not participate frequently in religious activities. In secularized societies, such as Denmark, the comparative importance of religious engagement may be lower than that of other components of social relations. In our study, we found a 2-fold increased risk of mortality when not attending clubs or organizations. Nevertheless, the effect was strongly attenuated after adjustment for demography, lifestyle and morbidity suggesting confounding by these factors. Findings were consistent with Pantell *et al*., although they only presented fully adjusted estimates^3^.

In the current study, we did not examine mediation and potential pathways from social isolation to mortality. However, several pathways have been proposed linking social relations to health and mortality including behavioral, psychological and biological factors^6,7,21^. Social isolation may influence health behaviors such as smoking, physical inactivity and alcohol use, which can increase mortality. Also, social relations can influence emotional states^22^, psychiatric comorbidity^23,24^ and a series of physiologic pathways largely related to stress responses, such as altered immune^25,26^ and neuroendocrine function^6^, which in turn may cause cardiovascular disease^27^.

According to the prior meta-analysis by Holt-Lunstad *et al*., data on the association between social isolation and mortality derive from North America (51%), Europe (37%) and Asia (11%)^1^. The association between social relations and mortality may be modified by various factors including cultural norms, distribution of socioeconomic status in the population, the social support system and health care system. Our study has confirmed the association between social isolation and increased mortality in a population with potential different confounding structures compared to prior studies conducted in high-income countries. Despite, the strong social system in Denmark, social isolation still appears to impact mortality with cohabitation status as an important predictor. This finding is in line with previous studies from Denmark or other Scandinavian countries with similar social support system as Denmark^8,10^. A study from Sweden found that low socio-economic position were associated with increased mortality after adjustment for demography and social network^9^. We found no effect of income (adjusted or stratified analyses) or education (adjusted analysis) in our study. The difference between studies may be explained by different methodology or different settings and confounding structures.

Our findings indicate that the effect of social isolation on mortality are similar to well-established risk factors such as alcohol consumption and obesity. Not having a spouse or a partner is an especially important risk factor. Yet, marriage rates are declining and an increasing portion of people is living alone^13^. Although important challenges remain in establishing that the link between social networks and mortality is causal, these major changes in the structure of social ties could have important consequences for population health.

Our study has several strengths. We conducted a population-based study and had full ascertainment of mortality, with virtually no misclassification. We were able to adjust for multiple potential confounders including underlying somatic and psychiatric morbidity, socioeconomic status and lifestyle factors. Our study also has limitations. First, the response rate in the survey was 69%, however, we accounted for non-response by post-survey weights in the regression models. Second, data were derived from participants’ self-reports and such data may not capture true levels of social activity and lifestyle. As example, a prior validation study of self-reported body weight and height in the NHANES questionnaire concluded that BMI tends to be underestimated using the self-reported data compared to objective measures^28^. Third, we constructed the SNI for this study, assigning the maximum possible number of contacts based on frequency responses to increase specificity of each component and the overall index. Nevertheless, misclassification of the social isolation variable is possible. Furthermore, we used variables judged to be equivalents of the ones used in the original SNI. Absence of exact equivalents could cause misclassification or correlation with other components. The SNI components describing partnership and religious activities are constructed from single targeted questions and are therefore likely to be the ‘cleanest’ components. Fourth, as lifestyle and morbidity were measured at baseline (cross sectional to each other), we were not able to disentangle if lifestyle precede morbidity or vice versa. This is often a general problem in studies of older adults, as covariates measured at baseline have probably been influencing one another dynamically for years or decades.

In conclusion, objective social isolation was associated with a 60–70% increased all-cause mortality, despite adjusting for several potential confounders, including psychiatric and somatic health status, lifestyle habits and socioeconomic status. Not having a spouse or a partner was associated with highest MRR in both genders. The importance of considering social relations as a factor for health should be acknowledges by the public and professionals. In addition, more research in this area to evaluate causality, and identify policies that could strengthen social integration or remediate the health consequences of isolation should be prioritized.

## Methods

The study population included participants in the ongoing cross-sectional Danish health survey (HHDD). Between February to May 2006, a random sample of 31,500 persons aged 25 to 79 years who resided in the Central Denmark Region and had at least one parent born in Denmark were invited to participate^14^. A total of 21,637 (69%) agreed to participate and completed a detailed questionnaire. The follow-up ended on the date of death, emigration, or 1 January 2013, whichever came first.

We used HHDD data to reconstruct the SNI^4^, replicating methodology of other studies that used the SNI^3,4,25^. The four dichotomized characteristics summarized by the SNI are marriage/partnership; frequency of social contacts; frequency of religious participation; and memberships in groups (e.g., clubs, associations). For the marriage/partnership variable, we assigned the value of 1 to participants who were married or in a steady partnership (cohabiting); otherwise 0. For the frequency of social contacts, we followed the methodology of Pantell *et al*.^3^ and assigned the value of 1 (frequent social contacts) to participants with greater than 156 contacts with friends or family annually (roughly, one contact every other day); otherwise 0. We calculated the frequency of social contacts from responses to the questions “How often are you in contact with friends and acquaintances (spending time together, talking on the telephone or writing)?”, “How often are you in contact with family you do not live with (spending time together, talking on the telephone or writing)?”, and “How often are you invited by others?”. The number of annual contacts for each of the questions was estimated from the maximum numeric value assigned to each of the multiple-choice response options: “daily or nearly daily” (365 contacts), “once or twice per week” (104 contacts), “once or twice per month” (24 contacts), and “rarely” (11 contacts) to the questions about contacts with friends or family. The total annual number of social contacts was the sum of the frequencies estimated from the responses to the three questions. We argue that assigning the maximum possible number of contacts to each response is correctly classifying socially isolated individuals using the HHDD data. For the frequency of religious participation variable, we used responses to the question “Have you in the past year participated in religious activities, including worship, in your spare time”? We assigned the value of 1 (frequent religious participation) to participants responding”daily”, “once or more per week”, “once or more per month”, or “approximately every other month”; we assigned the value of 0 to participants responding “rarely or never”. Finally, the frequent club membership factor was estimated based on responses to questions about frequency, in the previous year, of attending school or lectures; taking courses; participating in sports; singing in a choir; participating in school or day care board meetings; volunteering; socializing in community centers; and attending political or professional meetings. Participants responding “rarely or never” to the frequency of all of the listed activities were assigned the value of 0 on the group membership variable; otherwise they were assigned the value of 1. The overall SNI was the sum of the four components, thus ranging from 0 (most socially isolated) to 4 (least socially isolated). In accordance with other studies, we defined the categories of SNI as 0/1, 2, 3, and 4^3,4^.

We obtained data on potential confounders using self-reported information in the HHDD, inpatient and outpatient diagnoses recorded in the Danish National Registry of Patients (DNRP)^29^, and outpatient prescription dispensations, as recorded in Danish National Health Service Prescription database (DNHSPD)^30^ Self-reported variables included minimum years of education, personal annual income, smoking (never, former, current), level of alcohol use per week in Danish units (one Danish unit is defined as 15 milliliters or 12 gram of pure alcohol), height and weight; and self-reported health status. From self-reported height and weight, we calculated body mass index (BMI, kg/m^2^), analyzed in categories of <18.5 (underweight), 18.5 −<25.0 (normal, reference), 25.0 −<30 (overweight), and ≥30 (obese)^31^. From the DNRP, we linked diagnoses recorded during hospital encounters, summarized using the Charlson Comorbidity Index^32,33^. From the DNHSPD, we linked data on current and past prescriptions of antidepressants, and analgesics.

Information on deaths came from the Danish Civil Registration System, which is a total population registry tracking births, deaths and migrations, with daily updates^34^. This Registry issues a unique identifier to each Danish resident at birth or immigration. The identifier encodes date of birth and gender and is used universally, allowing individual-level record linkage of different data sources.

We cross-tabulated the distribution of the participants’ demographic, lifestyle and medical characteristics according to the categories of SNI. All subsequent analyses were stratified by gender. We calculated overall and SNI-category-specific cumulative mortality during the seven years of follow-up and constructed Kaplan-Meier survival curves for visualization, separately for men and women. We used Cox proportional-hazard regression (with time since completion of the questionnaire as time scale) to estimate, via hazard ratios, crude and adjusted mortality rate ratios (MRR) with 95% confidence interval (CI), comparing each of the SNI categories 0/1–3 against the reference SNI category 4. We fit several regression models based on covariates included as independent variables in addition to the SNI (as a categorical variable). Model 1 included age at survey completion, education level and personal income; Model 2 included the variables in Model 1 and lifestyle variables (body mass index, smoking, alcohol intake, and physical inactivity). Model 3 included the variables in Model 2 and morbidity variables (Charlson Comorbidity Index, use of antidepressants and analgesics [none, past, current]). We also fit Models 1–3 using the four SNI components as the independent variables. The assumption of proportional hazards was graphically verified.The analyses were conducted as complete-case analyses. In the regression models, we accounted for survey design and non-response by using post-survey weights computed at Statistic Denmark^35^.

We conducted multiple sensitivity analyses. First, we changed the order of which lifestyle and morbidity were introduced into model 2 and 3. In model 2, we adjusted for age, income, education and morbidity. In model 3, we additionally adjusted for lifestyle. Second, we stratified on income level (income below 149,000 Danish Kroners per year vs. income of 149,000 Danish Kroners per year or above) to evaluate effect measure modification by income level. Third, we evaluated reverse causation by excluding people with a high level of morbidity (Charlson Comorbidity Index >2) or people with terminally illness registered 1 year prior to index date.

Algorithms are provided in the Supplementary Tables S5 and S6.

All statistical analyses were performed using SAS (version 9.4; SAS Institute Inc, Cary, NC, USA).

This study was approved by the Danish Data Protection Agency (record no. 2013-41-1924). According to Danish legislation, this study does not require approval from the Ethics committee.

### Data availability

The datasets generated during and/or analysed during the current study are not publicly available due to Danish legislation.

## Electronic supplementary material

Supplementary Information
