# Supplementary material for: Social isolation and all-cause mortality: a population-based cohort study in Denmark
Source: Sci Rep. 2018 Mar 16;8:4731. doi: 10.1038/s41598-018-22963-w (PMC5856842; doi:10.1038/s41598-018-22963-w)
Supplement: Supplementary file 1 — Supplementary Information [file 41598_2018_22963_MOESM1_ESM.pdf]

Title: Social isolation and all-cause mortality: a population-based cohort study in Denmark

Kristina Laugesen,[1,*] Lisbeth Munksgård Baggesen,[1] Sigrún Alba Jóhannesdóttir Schmidt,[1] M. Maria Glymour,[2] Mathias Lasgaard,[3,4] Arnold Milstein,[5] Henrik Toft Sørensen,[1,6] Nancy E. Adler,[7] Vera Ehrenstein[1]

[1] Aarhus University Hospital, Department of Clinical Epidemiology, Aarhus, 8200, Denmark

[2] University of California, Department of Epidemiology and Biostatistics, San Francisco, CA, USA

[3] Central Denmark Region, DEFACTUM, Aarhus, 8200, Denmark

[4] University of Southern Denmark, Department of Psychology, Odense, 5230, Denmark

[5] Stanford University School of Medicine, The Clinical Excellence Research Center (CERC), Stanford, CA, USA

[6] Stanford University, Department of Health Research & Policy, Stanford, CA, USA

[7] University of California, Departments of Psychiatry and Pediatrics, San Francisco, CA, USA

[*] Kristina.laugesen@clin.au.dk

SUPPLEMENTARY

Supplementary Table S1. Distribution of social relations according to Social Network Index, stratified by gender

	Men				Women			
	Social Network Index				Social Network Index			
	0/1	2	3	4	0/1	2	3	4
	N	N	N	N	N	N	N	N
Marriage/partnership								
Missing	115	121	25	0	102	159	57	0
Married	420	2,038	3,505	954	289	1,743	4,209	1,307
Steady partnership	118	392	842	71	38	376	984	97
Alone/ Never married/ Never in a steady partnership	226	306	54	0	84	192	65	0
Widow/widower	69	70	35	0	139	331	240	0
Alone due to separation or divorce	143	198	34	0	165	336	92	0
Alone after termination of partnership	103	168	16	0	87	213	32	0
Frequency of contacts with friends								
Missing	47	45	26	7	39	99	69	12
Daily/almost daily	209	1,259	2,366	590	209	1,475	2,984	826
Once or twice per week	446	1,307	1,942	411	288	1,145	2,311	524
Once or twice per month	361	599	154	17	262	578	289	39
Less than once or twice per month	129	123	30	0	116	125	51	<5
Never	25	5	<5	0	13	8	<5	<5
Frequency of contacts with family								
Missing	49	43	12	<5	41	53	21	<5
Daily/almost daily	124	788	1,528	387	187	1,319	2,761	692
Once or twice per week	333	1,356	2,500	571	311	1,393	2,583	649
Once or twice per month	468	894	378	55	259	511	276	49
Less than once or twice per month	201	234	94	9	102	141	51	9
Never	42	23	7	<5	27	13	13	<5
Frequency of invites by others								
Missing	27	31	9	<5	27	40	24	5
Daily/almost daily	8	36	52	14	13	49	67	23
Once or twice per	109	655	1,348	397	117	845	1,767	549

	Men				Women			
	Social Network Index				Social Network Index			
	0/1	2	3	4	0/1	2	3	4
	N	N	N	N	N	N	N	N
week								
Once or twice per month	594	1,935	2,716	553	435	1,839	3,329	768
Less than once or twice per month	441	669	388	59	305	645	515	58
Never	38	12	6	0	30	12	<5	<5
Frequency of religious activities								
Missing	93	138	47	0	117	194	85	0
Daily	<5	<5	6	9	<5	<5	13	13
Once or more per week	0	17	68	161	0	20	111	214
Once or more per month	<5	19	122	321	<5	35	257	394
Approximately every second month	9	55	278	534	7	81	466	783
Rare or never	1,113	3,106	3,998	0	801	3,097	4,773	0
Frequency of attending school or lectures								
Missing	95	162	85	37	126	233	208	68
Daily	<5	12	19	13	0	25	40	10
Once or more per week	<5	38	127	24	5	63	158	51
Once or more per Month	0	31	76	22	<5	43	100	40
Approximately every second month	6	45	138	33	<5	49	140	48
Rare or never	1,111	3,050	4,075	896	789	3,017	5,059	1,187
Frequency of course attendence								
Missing	93	144	49	11	112	173	118	29
Daily	0	10	15	11	<5	9	21	10
Once or more per week	16	134	329	117	21	264	687	243
Once or more per month	9	104	331	200	14	163	533	292
Approximately every second month	16	207	570	210	18	329	895	318
Rare or never	1,083	2,739	3,225	476	760	2,492	3,451	512
Attendance at board meetings								
Missing	95	142	61	24	125	207	152	53
Daily	6	57	153	46	<5	34	75	23
Once or more per week	6	92	277	87	5	61	166	82
Once or more per month	21	274	728	253	10	215	679	252
Approximately	25	287	657	153	14	278	805	227

	Men				Women			
	Social Network Index				Social Network Index			
	**0/1**	**2**	**3**	**4**	**0/1**	**2**	**3**	**4**
	**N**	**N**	**N**	**N**	**N**	**N**	**N**	**N**
every second month								
Rare or never	1,064	2,486	2,633	462	771	2,635	3,828	767
Participating in sports								
Missing	100	187	132	51	130	255	287	89
Daily	7	91	167	33	7	92	234	47
Once or more per week	60	762	1,916	297	48	860	2,559	510
Once or more per month	18	188	418	110	13	171	478	140
Approximately every second month	7	89	194	61	6	96	245	76
Rare or never	1,025	2,021	1,692	473	723	1,956	1,902	542
Singing in choir								
Missing	92	134	48	14	117	184	103	33
Daily	0	0	10	9	0	6	13	6
Once or more per week	5	52	148	81	8	78	203	144
Once or more per month	<5	35	98	51	<5	36	108	64
Approximately every second month	<5	31	73	45	<5	27	93	65
Rare or never	1,116	3,082	4,142	825	794	3,099	5,185	1,092
Scouting								
Missing	98	150	87	25	121	213	174	55
Daily	<5	27	62	18	<5	22	39	18
Once or more per week	16	194	464	131	16	166	493	220
Once or more per month	8	106	300	115	12	107	386	177
Approximately every second month	6	88	235	75	<5	86	264	120
Rare or never	1,088	2,773	3,371	661	774	2,836	4,449	814
Socializing in community centers								
Missing	92	132	34	11	108	148	64	18
Daily	<5	13	11	7	<5	11	20	10
Once or more per week	19	157	335	95	23	220	462	136
Once or more per month	26	171	402	145	26	222	508	190
Approximately every second month	30	356	742	228	16	270	828	298
Rare or never	1,048	2,509	2,995	539	751	2,559	3,823	752
Attending political or professional meetings								
Missing	93	129	41	8	118	175	96	27
Daily	0	<5	9	5	0	<5	5	5
Once or more per	0	14	61	19	<5	15	31	14

	Men				Women			
	Social Network Index				Social Network Index			
	0/1	2	3	4	0/1	2	3	4
	N	N	N	N	N	N	N	N
week								
Once or more per month	6	61	215	84	5	66	152	71
Approximately every second month	23	230	516	201	14	146	432	162
Rare or never	1,095	2,901	3,677	708	789	3,026	4,989	1,125

Supplementary Table S2. Association between social isolation and all-cause mortality in Denmark, changing the sequence of covariate adjustment

	**Men**				**Women**			
	MRR (95% CI)				MRR (95% CI)			
	Crude	Model 1	Model 2	Model 3	Crude	Model 1	Model 2	Model 3
Social network index (SNI)								
0/1 vs 4	2.4 (1.7-3.5)	2.4 (1.6-3.5)	1.9 (1.3-2.9)	1.7 (1.1-2.6)	2.7 (1.8 -4.0)	3.0 (1.9-4.8)	2.2 (1.4-3.5)	1.6 (0.8-2.9)
2 vs 4	1.4 (0.98-1.9)	1.5 (1.1-2.2)	1.4 (0.97-2.0)	1.1 (0.74-1.7)	1.4 (0.96-2.0)	2.0 (1.3-3.1)	1.7 (1.1-2.5)	1.2 (0.73-2.1)
3 vs 4	0.95 (0.68-1.4)	1.3 (0.90-1.9)	1.3 (0.90-1.9)	1.3 (0.84-1.9)	0.77 (0.54-1.1)	1.3 (0.86-2.0)	1.2 (0.76-1.8)	0.98 (0.59-1.6)
SNI component (no vs. yes)								
Married/partner	1.4 (1.1-1.7)	1.5 (1.1-1.9)	1.5 (1.1-1.9)	1.5 (1.2-2.1)	2.9 (2.3-3.7)	2.1 (1.6-2.7)	2.0 (1.5-2.6)	1.7 (1.2-2.4)
Social contact	1.0 (0.82-1.3)	1.0 (0.81-1.3)	1.0 (0.82-1.3)	1.1 (0.82-1.4)	1.1 (0.83-1.6)	1.2 (0.88-1.7)	1.1 (0.75-1.5)	1.2 (0.72-1.9)
Religious activities	0.75 (0.58-0.98)	1.1 (0.80-1.4)	1.1 (0.80-1.5)	1.0 (0.73-1.4)	0.57 (0.44-0.74)	1.0 (0.79-1.4)	1.0 (0.76-1.4)	0.77 (0.52-1.1)
Clubs/organizations	2.4 (1.9-2.9)	1.6 (1.3-2.0)	1.4 (1.1-1.8)	1.2 (0.89-1.5)	1.9 (1.5-2.5)	1.6 (1.2-2.1)	1.3 (0.97-1.8)	1.1 (0.70-1.6)

Model 1: adjusted for age, education, and income

Model 2: adjusted for age, education, income, self-rated health, Charlson Comorbidity Index, past or present use of antidepressants, and past or present use of strong analgesics

Model 3: adjusted for age, education, income, self-rated health, Charlson Comorbidity Index, past or present use of antidepressants, and past or present use of strong analgesics, smoking, alcohol, body mass index, regular exercise

Supplementary Table S3. Association between social isolation and all-cause mortality in Denmark, stratified on low income and normal/high income

	**Low income[a]**				**Normal/High income[b]**			
	MRR (95% CI)				MRR (95% CI)			
	Crude	Model 1	Model 2	Model 3	Crude	Model 1	Model 2	Model 3
Social network index (SNI)								
0/1 vs 4	2.3 (1.7 – 3.1)	2.4 (1.8 – 3.4)	1.9 (1.3 – 2.8)	1.6 (1.0 – 2.4)	2.6 (1.7 – 3.9)	3.1 (2.0 – 4.6)	1.9 (1.2 – 3.0)	1.7 (1.1 – 2.7)
2 vs 4	1.5 (1.1 – 2.0)	1.8 (1.3 – 2.4)	1.4 (0.94 – 2.0)	1.3 (0.87 – 1.8)	1.3 (0.93 – 2.0)	1.8 (1.2 – 2.6)	1.3 (0.84 – 1.9)	1.3 (0.84 – 1.9)
3 vs 4	1.0 (0.75 – 1.4)	1.1 (0.84 – 1.5)	1.1 (0.75 – 1.5)	1.1 (0.74 – 1.5)	1.0 (0.69 – 1.5)	1.4 (0.95 – 2.0)	1.2 (0.82 – 1.8)	1.2 (0.83 – 1.8)
SNI component (no vs. yes)								
Married/partner	1.5 (1.3 – 1.9)	1.8 (1.5 – 2.2)	1.8 (1.4 – 2.3)	1.7 (1.3 – 2.1)	1.9 (1.5 – 2.5)	1.9 (1.5 – 2.4)	1.7 (1.3 – 2.2)	1.4 (1.1 – 1.9)
Social contact	1.4 (1.2 – 1.8)	1.3 (1.1 – 1.7)	1.3 (0.98 – 1.7)	1.3 (1.0 – 1.7)	0.90 (0.69 – 1.2)	0.88 (0.67– 1.2)	0.85 (0.63 – 1.1)	0.82 (0.61 – 1.1)
Religious activities	1.0 (0.80 – 1.2)	1.2 (0.94 – 1.4)	1.1 (0.84 –1.5)	1.1 (0.84 – 1.5)	0.70 (0.54 – 0.91)	0.99 (0.75 – 1.3)	0.87 (0.65 – 1.2)	0.88 (0.66 –1.2)
Clubs/organizations	1.4 (1.2 – 1.7)	1.2 (1.0 – 1.5)	0.99 (0.77 – 1.3)	0.87 (0.68 – 1.1)	2.4 (1.9 – 3.0)	2.1 (1.7 – 2.6)	1.6 (1.2 –2.1)	1.5 (1.2 – 2.0)

[a] Income below 149,000 Danish Kroners per year
[b] Income above 149,000 Danish Kroners per year
Model 1: adjusted for age and education
Model 2: adjusted for age, education, smoking, alcohol, body mass index, and regular exercise
Model 3: adjusted for age, education, smoking, alcohol, body mass index, regular exercise, self-rated health, Charlson Comorbidity Index, past or present use of antidepressants, and past or present use of strong analgesics

Supplementary Table S4. Association between social isolation and all-cause mortality in Denmark, excluding persons registered terminal ill or with high level of comorbidity (Charlson Comorbidity Index > 2) 1 year prior to index date

	**Men**				**Women**			
	MRR (95% CI)				MRR (95% CI)			
	Crude	Model 1	Model 2	Model 3	Crude	Model 1	Model 2	Model 3
Social network index (SNI)								
0/1 vs 4	2.5 (1.7-3.6)	2.4 (1.6-3.6)	2.0 (1.3-3.2)	1.7 (1.1-2.8)	2.8 (1.9-4.2)	3.3 (2.1-5.3)	2.1 (1.2-3.8)	1.7 (0.90-3.2)
2 vs 4	1.3 (0.94-1.9)	1.5 (1.0-2.2)	1.2 (0.77-1.8)	1.2 (0.76-1.8)	1.5 (1.0-2.2)	2.3 (1.5-3.5)	1.6 (0.94-2.7)	1.5 (0.90-2.6)
3 vs 4	0.94 (0.66-1.3)	1.3 (0.88-1.9)	1.2 (0.81-1.8)	1.2 (0.82-1.9)	0.81 (0.56-1.2)	1.4 (0.92-2.2)	1.1 (0.67-1.9)	1.1 (0.67-1.9)
SNI component (no vs. yes)								
Married/partner	1.4 (1.1-1.8)	1.6 (1.2-2.0)	1.6 (1.2-2.2)	1.6 (1.2-2.2)	3.1 (2.4-4.0)	2.3 (1.7-2.9)	2.2 (1.6-3.0)	1.9 (1.2-2.7)
Social contact	1.1 (0.85-1.4)	1.1 (0.84-1.4)	1.1 (0.81-1.4)	1.1 (0.82-1.4)	1.1 (0.8-1.6)	1.2 (0.86-1.7)	1.3 (0.85-1.9)	1.1 (0.67-1.8)
Religious activities	0.72 (0.55-0.94)	1.0 (0.77-1.4)	1.0 (0.71-1.4)	1.0 (0.71-1.4)	0.60 (0.45-0.78)	1.1 (0.82-1.5)	0.82 (0.57-1.2)	0.75 (0.51-1.1)
Clubs/organizations	2.3 (1.9-2.9)	1.6 (1.3-2.0)	1.4 (1.0-1.8)	1.2 (0.93-1.6)	1.9 (1.4-2.5)	1.5 (1.1-2.1)	1.2 (0.86-1.8)	1.1 (0.75-1.7)

Model 1: adjusted for age, education, and income
Model 2: adjusted for age, education, income, smoking, alcohol, body mass index, and regular exercise
Model 3: adjusted for age, education, income, smoking, alcohol, body mass index, regular exercise, self-rated health, Charlson Comorbidity Index, past or present use of antidepressants, and past or present use of strong analgesics

Supplementary Table S5. Covariates listed with their categories, their *International Classification of Diseases,* 8th revision (ICD-8) codes, their *International Classification of Diseases,* 10th revision (ICD-10), their anatomical therapeutic chemical (ATC) classification codes or based on the "How are you?" (HHDD) questionnaire.

Covariates	ICD-8 codes	ICD-10 codes	ATC codes	Answers to HHDD questionnaire
Hypertension (ever before baseline)	400-404	I10-I15		'Yes, I have it now' or 'yes, I have previously had it'
COPD, bronchitis, emphysema (ever before baseline)	491-492	J41-44		"Yes, I have it now", or "yes, I had it in the past"
Diabetes (ever before baseline)			A10	"Yes, I have it now", or "yes, I had it in the past"
Dyslipidemia (ever before baseline)			C10, B04AA10, BA04AA24, B04AA25, B04AC01-10, B04AB01-B04AB04, B04AD01-03, B04AE01-05, B04AX01-03, and B04AX06-09	
Antidepressants (ever before baseline)			N06A (excluding N06AX12 from 2009 and thereafter)	
Strong analgesics (ever before baseline)			N02A	
Terminally illness		Z515, Z756 or supplementary code ZNAC14, ZPZA05		

Supplementary Table S6. Algorithm for Charlson Comorbidity Index containing International Classification of Diseases, 10th revision

	Diseases[a]	ICD-10	Score
1	Myocardial infarction	I21;I22;I23	1
2	Congestive heart failure	I50; I11.0; I13.0; I13.2	1
3	Peripheral vascular disease	I70; I71; I72; I73; I74; I77	1
4	Cerebrovascular disease	I60-I69; G45; G46	1
5	Dementia	F00-F03; F05.1; G30	1
6	Chronic pulmonary disease	J40-J47; J60-J67; J68.4; J70.1; J70.3; J84.1; J92.0; J96.1; J98.2; J98.3	1
7	Connective tissue disease	M05; M06; M08; M09; M30; M31; M32; M33; M34; M35; M36; D86	1
8	Ulcer disease	K22.1; K25-K28	1
9	Mild liver disease	B18; K70.0-K70.3; K70.9; K71; K73; K74; K76.0	1
10	Diabetes type1	E10.0, E10.1; E10.9	1
	Diabetes type2	E11.0; E11.1; E11.9	
11	Hemiplegia	G81; G82	2
12	Moderate to severe renal disease	I12; I13; N00-N05; N07; N11; N14; N17-N19; Q61	2
13	Diabetes with end-organ damage type1 and type2	E10.2-E10.8<br>E11.2-E11.8	2
14	Any tumor (except BC)	C00-C75 (excluding C64)	2
15	Leukemia	C91-C95	2
16	Lymphoma	C81-C85; C88; C90; C96	2
17	Moderate to severe liver disease	B15.0; B16.0; B16.2; B19.0; K70.4; K72; K76.6; I85	3
18	Metastatic solid tumor	C76-C80	6
19	AIDS	B21-B24	6

[a] Diagnosis were assessed up to five years before filling in the “How are you” questionnaire
